# A mobile health-based lifestyle intervention on gestational weight gain in pregnant women with overweight or obesity: protocol of a randomized controlled trial (the HGWG-pro study)

**DOI:** 10.1186/s12884-025-08595-9

**Published:** 2025-12-27

**Authors:** Haixue Wang, Ping Li, Lan Cheng, Xin Yuan, Jingjing Wang, Yuge Zhang, Qi Ye, Yingying Mou, Qinyi Ma, Haijun Wang

**Affiliations:** 1 Department of Orthopaedics, Peking University Third Hospital, Beijing, China; 2 Department of Maternal and Child Health, School of Public Health, Peking University, Beijing, China; 3 Peking University Health Science Center-Weifang Joint Research Center for Maternal and Child Health, Beijing, China; 4 MRC Centre for Environment and Health, Department of Epidemiology and Biostatistics, School of Public Health, Imperial College London, London, W12 0BZ UK; 5 China Institute of Sport Science, Physical Fitness and Scientific Exercise Research Center, Beijing, China; 6 Shandong Second Medical University, Shandong Weifang, China; 7 Tongzhou Maternal and Child Health Care Hospital of Beijing, Beijing, China; 8 Weifang Maternal and Child Health Hospital, Shandong Weifang, China; 9 Department of Epidemiology and Biostatistics, School of Public Health, Peking University, Beijing, China

**Keywords:** Overweight or obesity, Pregnancy, Gestational weight gain, Lifestyle intervention, Mobile health

## Abstract

**Background:**

Excessive gestational weight gain (EGWG) among women with overweight or obesity is a public health concern, increasing the risk of adverse pregnancy outcomes. Although lifestyle intervention has been proved effective on EGWG control, few successful mobile health-based lifestyle interventions exist in healthcare settings.

**Methods:**

The Healthy Gestational Weight Gain program (HGWG-pro) is a two-centered randomized controlled trial that aims to evaluate the effectiveness of a multi-component intervention on promoting appropriate gestational weight gain, preventing gestational diabetes mellitus (GDM), and improving pregnancy, delivery, and neonatal outcomes among overweight or obese pregnant women aged 18 to 40 years old in China. Two maternal and child health care hospitals will be selected from the eastern region of China. Altogether 200 women with pre-pregnancy body mass index (BMI) ranging from 24 to 40 kg/m^2^ from the two hospitals will be randomized to intervention or control group. The intervention will be delivered from 10^+ 0^-13^+ 6^ weeks’ gestation to 32^+ 0^–35^+ 6^ weeks of gestation, with biweekly counseling sessions conducted either face-to-face during routine prenatal visits or via telephone, depending on the participant’s schedule. A smartphone application and wearable smart wristband will be used to assist in providing GWG related information, monitoring dietary behaviors, physical activity and weight, and providing feedback on weight and behaviors for participants. Data will be collected at 8^+ 0^-13^+ 6^, 24^+ 0^-27^+ 6^, and 32^+ 0^-35^+ 6^ weeks’ gestation, at delivery, at 42 days, 6 months and 12 months after delivery. This study has two primary outcomes: the between-group differences in gestational weight gain at 24^+ 0^-27^+ 6^ weeks’ gestation and 32^+ 0^-35^+ 6^ weeks’ gestation. The secondary outcomes will include differences between groups in weekly rate of GWG and incidence of adverse pregnancy complications and pregnancy outcomes, changes in diet, physical activity and other offspring health indicators at follow-up visits. Metabolic blood test results including indicators such as blood sugar and blood lipids will be extracted from the electronic medical record (EMR) system, indicators such as insulin and glycated hemoglobin will be measured through the collected blood samples. This trial includes a variety of process evaluation methods to evaluate the fidelity and adherence of the intervention.

**Discussion:**

The HGWG-pro is one of the few mobile health-based interventions targeting EGWG among women with overweight or obesity in China. This multi-component intervention study is unique in its assessment of long-term maternal and infant health outcomes, extending up to 12 months postpartum, and the intervention methods based on mobile health contribute to yield an intervention model suitable for widespread application in healthcare settings.

**Trial registration:**

ClinicalTrials.gov (NCT06370533): registered on 31th March 2024.

## Background

Excessive gestational weight gain (EGWG) has been a public health concern, with nearly half of pregnant women gaining more gestational weight gain than the Institute of Medicine (IOM) guideline [1]. Women with overweight or obesity before pregnancy are more easily suffering from EGWG than normal-weight women [1–3]. Overweight or obesity before pregnancy itself can increase the risk of pregnancy and delivery complications as well as future obesity, diabetes, and cardiovascular risk [4, 5], while EGWG can further amplify this adverse effect [6, 7]. Thus, it’s of great significance to conduct intervention studies among women with pre-pregnancy overweight or obesity.

Lifestyle intervention is a cost-effective strategy that has demonstrated significant effect on improving gestational weight gain (GWG) [8–10]. Evidences indicated that intensive behavioral lifestyle interventions with in-person counselling were effective in preventing EGWG among overweight or obese women [11, 12]. The Gestational Weight Gain and Optimal Wellness (GLOW) trial introduced telephone-based lifestyle intervention to make the intensive behavioral lifestyle interventions become feasible in healthcare settings and achieved success [13]. However, except that physical activity was evaluated by accelerometer objectively in the GLOW study, other self-monitoring (like weight and diet) and dietary goal setting were performed by traditional ways without the assistance of mobile health.

The emergence of mobile health opens the way for innovative behavioral change intervention approaches. However, a few lifestyle interventions based on mobile health existed and not all of them were effective on GWG control among women with overweight or obesity [14]. Due to the various intervention design across studies, the optimal intervention approach based on mobile health technology (including intervention components, delivery modes and behavioral strategies) is still unknown [15]. Still, many of their dietary intervention targeting low glycemic index (GI) or energy control, which are complex for both women and health care providers [16–18]. In addition, evidence regarding the long-term effects of intervention on maternal and infant health beyond the pregnancy and immediate postpartum period are needed [19]. Thus, based on the components of an efficient GLOW trial and usual antenatal care process in China, we developed the mobile health-based lifestyle intervention, Healthy Gestational Weight Gain program (HGWG-pro). The study was designed to be feasible in healthcare settings with the aim of evaluating the effectiveness of a multicomponent lifestyle intervention compared with the usual antenatal care in preventing EGWG among women with overweight or obesity.

## Methods

### Study design

The HGWG-pro study is a two-center randomized controlled trial. To examine the universality of the intervention in cities with different socio-economic levels in the eastern area of China with a high prevalence of obesity [20], we will select two hospitals from different regions of China: Beijing, whose per-capita GDP is the highest among the 10 eastern provinces, and Shandong, whose per-capita GDP ranks eighth among the ten eastern provinces [21]. Tongzhou Maternal and Child Health hospital and Weifang Maternal and Child Health Hospital will be selected in this program. The process of routine prenatal care in the two hospitals is similar, except that Tongzhou Maternal and Child Health hospital operates a nutrition clinic where all pregnant women are suggested to visit. They are provided with a 20-minute health education session focusing on the essentials of a healthy pregnancy. Furthermore, early-stage pregnant women with a body mass index (BMI) of 28 kg/m² or higher are offered an additional personalized nutrition consultation to support their health during pregnancy. However, this process does not affect our recruitment of participants because all those pregnant women who received the nutrition consultation will be randomly assigned to the intervention or the control group, and the nutrition clinic consultation belongs to the usual care of the hospital, which might not influence the intervention.

**Fig. 1:**
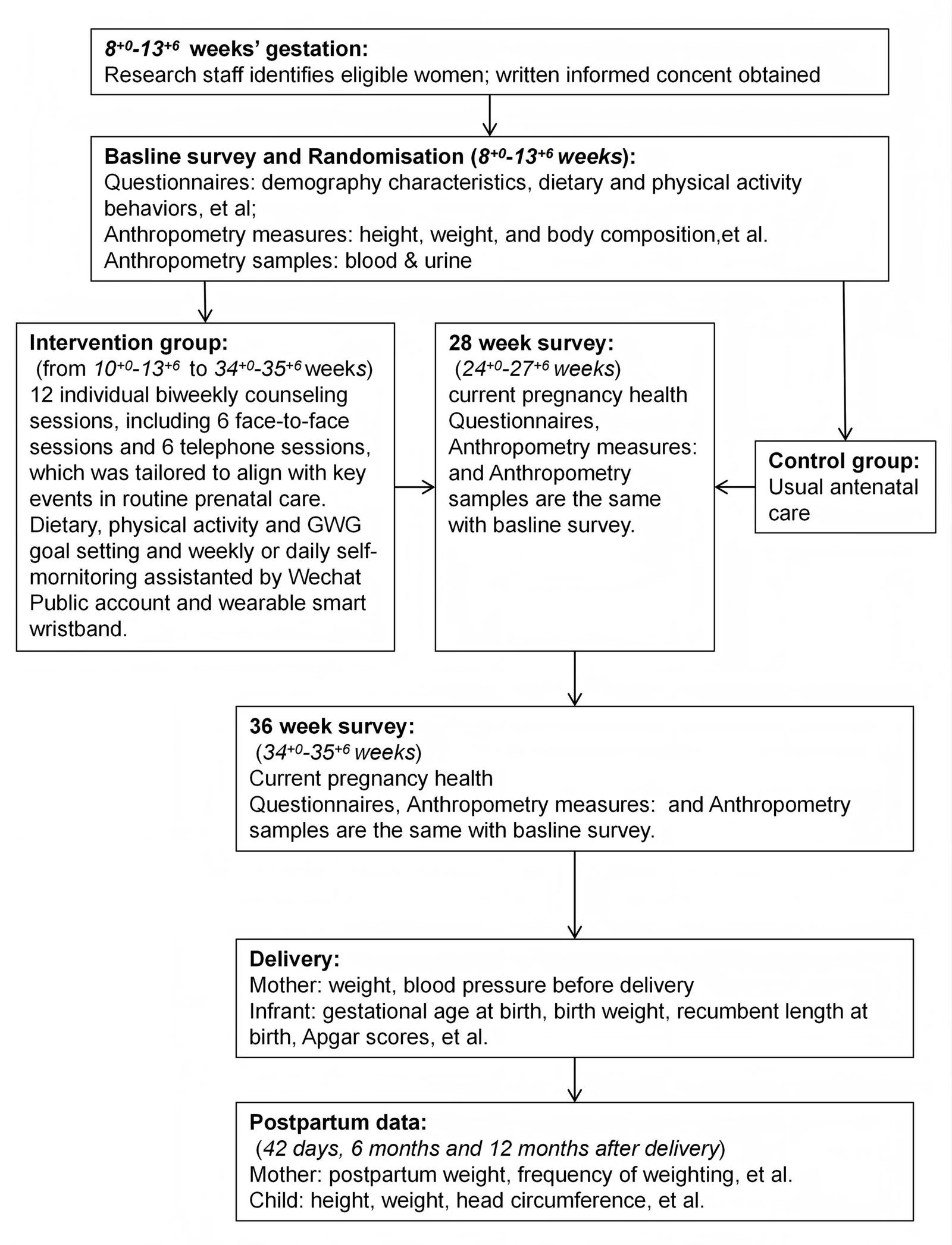
Flow chart of the HGWG-pro study

The intervention will last for approximately 6 months from 10^+ 0^−13^+ 6^ to 32^+ 0^−35^+ 6^ weeks’ gestation, and it will be implemented from April 2024 to February 2025, according to the time of last enrollment of women in the intervention group. The study will continue to delivery, 42 days, 6 months and 12 months after delivery with collection of delivery information and anthropometric measures. Figure 1 is the flow chart of the study.

### Eligibility and recruitment

The HGWG-pro study will recruit 200 overweight or obese pregnant women from 2 hospitals, with 100 participants from each hospital. Overweight or obesity were determined based on BMI recommendations of the Group of China Obesity Task Force of the Chinese Ministry of Health (overweight: 24 ≤ BMI < 28 kg/m^2^; obese: BMI ≥ 28 kg/m^2^) [22]. Pregnant women receiving usual antenatal care at the two hospitals will be firstly identified through gestational weeks. Only those at 8–14 weeks’ gestation will be further evaluated according to the inclusion criteria: aged between 18 and 40 years, pre-pregnancy BMI between 24 and 40 kg/m^2^ based on weight self-reported and recorded in the electronic medical record (EMR), singleton pregnancy, skilled at using smartphones and WeChat (mobile application), planning to attend regular antenatal care and delivery at Weifang Maternal and Child Health Hospital of Shandong or Tongzhou Maternal and Child Health hospital of Beijing, no contraindications to physical activity according to Physical Activity Readiness Questionnaire (PAR-Q), willing to be randomized and cooperate with regular follow-up visits and sign informed consent.

Exclusion Criteria including women with pre-pregnancy hypertension, severe cardiovascular or cerebrovascular diseases, respiratory disease, hepatic or renal disease, malignant tumors, systemic lupus erythematosus, thyroid disease, severe anemia, and other chronic diseases, cervical insufficiency, multiple pregnancy, continuous vaginal bleeding, diabetes before pregnancy, special dietary needs (e.g. vegetarianism), severe psychiatric disorders, with a history of bariatric surgery or other surgical history involving important organs within 3 months; participated in other clinical trials within 6 months.

### Sample size estimation

The sample size was estimated to ensure adequate power for detecting group differences in GWG at both mid- (24^+ 0^−27^+ 6^ weeks) and late-pregnancy (32^+ 0^−35^+ 6^ weeks). The anticipated differences between the two groups were 2.19 kg [13] at 32^+ 0^−35^+ 6^ gestational weeks, and 1.19 kg at 24^+ 0^−27^+ 6^ gestational week [23], the SD of the gestational weight gain would be 4 kg and 2 kg at both mid- and late-pregnancy, respectively. The rate of attrition would be 10% in each group. We aimed to recruit a total of 200 pregnant women from 2 hospitals, with 100 women per hospital. The sample size will provide 80% power with α = 0.05 to detect a mean difference of 2.19 kg in the gestational weight gain between groups after the intervention from 10^+ 0^ to 13^+ 6^ gestational weeks to 34^+ 0^~35^+ 6^ gestational weeks.

### Randomization

The study will obtain informed consent at the baseline clinic visit at 8^+ 0^ to 13^+ 6^ weeks’ gestation. Only those women who meet the inclusion criteria, consent and complete the baseline assessment will be randomly assigned to either intervention or control group.

The 100 participants from each hospital will be randomly allocated at a 1:1 ratio to either the intervention group or the control group by the study staff, stratified by the categorical variables of age (< 30 and ≥ 30 years), pre-pregnancy BMI (< 28 and ≥ 28 kg/m^2^), and history of delivery with randomly determined block sizes of 4. Neither the staff nor the participants will be blinded to the group assignment due to the nature of the intervention program. The process of the randomization will be supervised by another study staff and recorded in a Microsoft EXCEL database.

### Control group

Pregnant women randomized to the control group in the two hospitals will not receive any of the HGWG-pro intervention components and will continue their routine antenatal care according to the local hospital’s health care provision.

### Intervention

#### Intervention components

We used the transtheoretical model and stages of change (TTM) to guide the intervention strategies in this multi-component lifestyle intervention program. The intervention will be delivered by two trained members of the project through 12 individual biweekly counseling sessions, including 6 face-to-face sessions and 6 telephone sessions, which were tailored to align with key events in routine prenatal care.

The initial 40-minute face-to-face session (10^+ 0^ to 13^+ 6^ weeks) will cover the following topics: definition of EGWG and its adverse effects on maternal and child health, the recommended gestational weight gain based on Chinese references (Table 1) [24], dietary recommendations from the Dietary Guidelines for Chinese Residents [25],“two not, six less” (healthy eating principles: not eating excessively, not drinking sugar-sweetened beverages, eating less fried food, eating less unhealthy snacks, eating less Western-fast food, eating less takeout, eating less high-sugar desserts, and eating less processed meat), weekly physical activity and weight management goals for pregnancy. The biweekly sessions will be performed through telephone or face to face to provide feedback on behavioral changes and weight gain over the past two weeks based on data from the App and wristband, to review behavioral and gestational weight gain goals, and for participants with poor weight control, a detailed discussion of diet and exercise habits to identify key challenges will be provided, followed by tailored guidance.

**Table 1:** Recommended gestational weight gain standards for Chinese pregnant women

Pre-pregnancy BMI Category	Total Gestational Weight Gain	1 st Trimester Weight Gain	2nd & 3rd Trimester Weekly Gain
Underweight (BMI < 18.5 kg/m^2^)	11.0 ~ 16.0 kg	0 ~ 2.0 kg	0.46 kg (0.37 ~ 0.56)
Normal weight (18.5 ≤ BMI < 24.0 kg/m^2^)	8.0 ~ 14.0 kg	0 ~ 2.0 kg	0.37 kg (0.26 ~ 0.48)
Overweight (24.0 ≤ BMI < 28.0 kg/m^2^)	7.0 ~ 11.0 kg	0 ~ 2.0 kg	0.30 kg (0.22 ~ 0.37)
Obese (BMI ≥ 28.0 kg/m^2^)	5.0 ~ 9.0 kg	0 ~ 2.0 kg	0.22 kg (0.15 ~ 0.30)

The intervention will be assisted by mobile health apps and wearable devices. The pregnant women in the intervention group will be equipped with a weight management platform account, a smart wristband (Huawei Wristband 8), a weighing scale, and an Elastic band. Participants will receive in-person training on how to utilize the weight management App of the WeChat Public Account “Population health service platform” and the Wristband. These tools will enable them to access health education resources, track weight gain, monitor dietary behaviors and physical activity, and review feedback about their weight and lifestyle behaviors. Details about the intervention are described in Table 2.

**Table 2:** Key intervention components based the smartphone app with the help of wristband

Intervention components	Content
Health education activities	48 articles about weight management during pregnancy are presented in the health education module of the Wechat app, participants are required to read 2 to 4 articles per week from 10^+ 0^ to 35^+ 6^ gestational weeks.
Dietary behavior modification	(1) Dietary goal setting: 8 core dietary goal will be set by the program member according to women’s change intention. The eight eating behavioral goals will be assigned to each participant with two for every 4-week cycle. (2) Dietary behavior monitoring: women will be asked to record the 8 dietary behaviors in the App every week.
Active physical activity	(1) Goal reminding: 150 min MVPA per week, recommending 30 min of aerobic exercise (including brisk walking) 3 times a week and 30 min of resistance exercise 2 times a week; goal of 6000 steps/day) will be shown at the face of the physical activity module. (2) Exercise video providing: a 5 min of Warm-up exercises video, a 30 min of aerobic exercise video and a 30 min of resistance exercise video are provided for women to use. (3) Exercise monitoring: on the one hand, women will be asked to record the physical activity behaviors manually in the app every day, with the function of recording multiple times a day; on the other hand, women’s physical activity will be monitored by wearing a wristband every day, with the function of monitoring heart rate, steps, total physical activity time and different levels of physical activity time. (4) Safety reminding: Articles about how to exercise safety and how to deal with the muscle soreness caused by exercise are provided.
Weight management	(1) Weight gain goal reminding: weight gain goal per week/during the whole pregnancy and current total weight gain will be displayed at the top of the weight record page. (2) Weight monitoring: participants will be asked to record their weight at least one time per week, with the weighing condition of fasting in the morning, emptying urine and feces, and wearing light clothes. All participants in the intervention group will be provided with a weighing scale. (3) Feedback about weight gain: as soon as participants input the current weight, the platform will immediately pop up feedback about weight gain by comparing with last week’s weight.
Feedback	Tailored feedback in the form of graphical illustration and text according to diet, physical activity, and weight data collected by WeChat public account and a wristband. In the assessment feedback interface, participants’ weekly feedback report will be displayed from the following aspects: (1) Weight gain: Your pre-pregnancy weight was XX kg, your initial weight was XX kg, and your weight last week was XX kg. You measured your weight a total of XX times in this week, and your most recent weight is XX kg. Feedback for Good Weight Control (Positive Encouragement): Your weight control during this period has been excellent. Great job! Keep it up! Continue to maintain healthy eating and exercise habits, as your efforts are already showing results. The future looks bright! Feedback for Excessive Weight Gain During Pregnancy (Improvement Suggestions): It appears that your weight gain during pregnancy has been excessive. I suggest focusing on improving your diet and exercise behaviors in the coming weeks. Please weigh yourself weekly and keep a record, and maintain healthy lifestyle habits to ensure a healthy weight gain. Feedback for Rapid Weight Gain During Pregnancy (Improvement Suggestions): Your rate of weight gain during pregnancy has been relatively fast. I recommend paying more attention to your diet and exercise habits. Please weigh yourself weekly and keep a record, and continue to maintain healthy lifestyle habits to regulate the rate of weight gain. (2) Dietary behavior: Feedback based on the pregnant woman’s performance this week and comparison with last week: This week’s performance is good, and it’s as good as last week’s performance. Keep up the great work!/This week’s performance is good, and compared to last week, there has been improvement. Keep up the effort!/This week’s performance is poor, but compared to last week, there has been improvement. Keep pushing!/This week’s performance is poor, and there has been no improvement compared to last week. Put in more effort!/This week’s performance is poor, and there has been a setback compared to last week. You must put in more effort! (3) Physical activity behavior from the record: This week, you recorded exercising XX times, with a total of XX minutes of activity. You have met/failed to meet the goal of 150 min of weekly activity (based on the recorded activity time compared to the target of 150 min). Please keep up the good work. For the health of both you and your baby, make sure to walk more and move more. Don’t forget to record your activity after each exercise session!” (4) Physical activity data from the wristband: This week, your average daily activity distance was XX meters, with XX minutes spent walking, XX minutes spent running, and XX minutes spent on moderate to high-intensity activities. The average calories you burned each day was XX kcal. Your average daily step count was XX steps, and walking at least 6,000 steps per day is beneficial to your health. You achieved the goal on XX days this week (days with ≥ 6000 steps). Maintaining good exercise habits helps keep your body in a healthy state, so please keep up the great work! (5) Heart rate from the wristband: Based on the heart rate data from the wristband, provide feedback on the pregnant woman’s average heart rate and the duration of time spent within the target heart rate. It is recommended that the pregnant woman increase moderate exercise. (6) Sleep quality: Based on the sleep data from the wristband, provide feedback on the sleep situation and recommend 7–9 h of sleep duration (7) Intervention compliance scores: This week, you earned XX points, bringing your total to XX points. Following the intervention plan set by the project team and maintaining a good lifestyle will lead to more health benefits. Keep it up!”
Record reminder	women in the intervention group will receive a reminder to fill in the dietary behavior per week, physical activity behavior per day and weight monitoring results per week.

*MVPA* moderate-to vigorous-intensity physical activity, which was defined as exercise during which the heart rate of participant reaches reaches 40%-60% of the maximum heart rate (220- age[year])

All women who agreed to participate in this study will be enrolled in the online system associated with our WeChat public account. They will be assigned a unique ID. All those services are designed and implemented to ensure the confidentiality of participants ‘information, and data will be used only by project members who have signed a confidentiality agreement.

### Data collection

Data will be collected at clinic visits in the hospital where participants receive their usual care, and from the EMR system. Table 3 describes the study outcomes of the key three research appointments, including when and how those outcomes will be evaluated. The baseline assessment will be conducted at approximately 10 weeks’ gestation (between 8^+ 0^ and 13^+ 6^ weeks) from April to August 2024 for both the intervention and the control groups. Follow-up assessments will be conducted at approximately 26 weeks’ gestation (between 24^+ 0^ and 27^+ 6^ weeks); 34 weeks’ gestation (between 32^+ 0^ and 35^+ 6^ weeks); delivery (within 1 week after delivery); 42 days postpartum (6–8 weeks after delivery); 6 months postpartum (24–32weeks after delivery) and 12 months postpartum (48–56 weeks after delivery). Two well-trained key members of the project will be responsible for the collection, supervision, and management of data.

**Table 3:** Outcome measurements for the HGWG-pro study

Outcomes	8^+ 0^−13^+ 6^ weeks’ gestation	24^+ 0^−27^+ 6^ weeks’ gestation		32^+ 0^−35^+ 6^ weeks’ gestation		Device (Manufacturer, model)		Methods
Anthropometric measures								
Height	√					Upright Posture Height and Weight Measuring Instrument (Shanghe Technology SH-200)		Measured to the nearest 0.5 cm at least twice without wearing shoes.
Weight	√	√		√		Upright Posture Height and Weight Measuring Instrument (Shanghe Technology SH-200)		Measured to the nearest 0.1 kg at least twice through wearing light-weight clothing without shoes.
Systolic and diastolic blood pressures	√	√		√		Fully Automatic Electronic Blood Pressure Monitor (Kentairou HBP-9020)		Measured to the nearest 1 mm Hg at least twice
Body composition	√	√		√		Bioelectrical impedance (BIA) TANITA MC-780MA		According to the standard procedure
LMP and Gestational age	√							Confirmed by ultrasounds conducted before the baseline survey clinic visit and extracted from EMR
Pre-pregnancy weight	√							Self-reported by women and extracted from EMR, if pre-pregnancy weight is not available, the earliest pregnancy weight measured before 10 weeks’ gestation will be obtained.
Behavioral measures and other measures								
Attitude and knowledge about GWG and weight management	√	√		√		Attitude and knowledge about gestational weight gain and weight management measured by 8 items, which was designed based on published articles [26, 27]. For example, women’s perceived weight category, realization about the importance of knowing recommended weight gain during pregnancy, knowledge about the best amount of weight to gain in pregnancy, dangers of excess weight gain for mother and child, et al. Corresponding options will be set according to the content of the question. Women could finish the questionnaire at the clinic or take it home.		
Food intake	√	√		√		Dietary intake will be measured by food frequency questionnaire (FFQ) investigating the frequency of food consumption over the past one month[28]. Women should finish the questionnaire under the guidance of the trained staff.		
Dietary behavior	√	√		√		Dietary behavior will be measured by self-designed questionnaire to achieve the frequency of unhealthy dietary behaviors and it was designed according to our health education goals based on published articles [29, 30]. It includes the frequency of overeating, sugary beverages, fried foods, unhealthy snacks, Western fast food, ordering takeout, high-sugar desserts, and processed meats.		
Physical activity	√	√		√		Physical activity will be measured by International Physical Activity Questionnaire-Short Form (IPAQ-SF) [31]. It asks about the frequency and duration of various types of physical activities over the past 7 days. The metabolic equivalent (MET) for different types of activities will be calculated as below and the higher score means the higher physical activity level: walking MET-minutes/week = 3.3 × walking minutes × walking days; moderate MET-minutes/week = 4.0 × moderate-intensity activity minutes × moderate-intensity activity days; vigorous MET-minutes/week = 8.0 × vigorous-intensity activity minutes × vigorous-intensity activity days; total physical activity MET-minutes/week = walking + moderate + vigorous MET-minutes/week.		
Sleep quality	√	√		√		Sleep quality will be measured by Pittsburgh Sleep Quality Index (PSQI) [32]. The score ranges from 0 to 21 and the higher score indicates the higher sleep quality.		
Quality of life	√	√		√		Quality of life will be measured by 12-Item Short Form Survey (SF-12) [33]. The score ranges from 0 to 100 and the higher score indicates the higher quality of life.		
Depression	√	√		√		Depression will be measured by 9-item Patient Health Questionnaire (PHQ-9) [34, 35]. The score ranges from 0 to 27 and the higher score indicates higher level of depression. A score greater than 4 indicates depression.		
Anxiety	√	√		√		Anxiety will be measured by Zung’s Self Rating Anxiety Scale (SAS) [36]. The score ranges from 25 to 100 and the higher score indicates higher level of anxiety. A score greater than 49 indicates anxiety.		
Social support	√					Social support will be measured by Maternity Social Support Scale (MSSS)[37], the score ranges from 5 to 30 and the higher score indicates higher level of social support.		
Self-efficacy	√	√		√		Self-efficacy will be measured by General Self-Efficacy Scale (GSES) [38, 39]. The score ranged from 10 to 40 and the higher score indicates higher level of self-efficacy.		
The stage of gestational weight control behavior	√	√		√		The investigators will use two items to assess it. “Have the participants started to adjust their diet/physical activity to control their weight during pregnancy?“, “No and I do not plan to do so in the next 6 months/No, but I am ready to start within the next 6 months/No, but I am ready to start in the next 30 days/Yes, but not regularly/Yes, I am currently controlling my weight by adjusting my diet/physical activity regularly, but it has not reached 6 months/Yes, I have been controlling my weight for more than 6 months by adjusting my diet/physical activity” choices will be provided.		
Clinical outcomes								
Gestational Diabetes Mellitus (GDM)		√				75 g 2-hour oral glucose tolerance test (OGTT), Fasting blood, Venous blood diagnosed according to International Association of Diabetes and Pregnancy Study Group (IADPSG) criteria: Fasting plasma glucose (FPG) ≥ 5.1 mmol/L, and/or 1-h blood glucose ≥ 10.0 mmol/L, and/or 2-h blood glucose ≥ 8.5 mmol/L		
Preeclampsia/ Gestational hypertension		√		√		Preeclampsia was defined according to the criteria of the American College of Obstetricians and Gynecologists (ACOG) i.e. systolic blood pressure of ≥ 140 mmHg or diastolic blood pressure of ≥ 90 mmHg occurring after 20 weeks of gestation in a previously normotensive woman combined with new-onset proteinuria of ≥ 0.3 g/24 h. Gestational hypertension was defined similarly but without the presence of proteinuria.		

At the baseline and all follow-up visits before delivery, anthropometric measures including height, weight, systolic and diastolic blood pressures, and body composition will be collected by the trained members of the project using the same device according to the standard methods. The date of the last menstrual period (LMP) and gestational age will be confirmed by ultrasounds conducted before the baseline clinic visit and extracted from EMR. Pre-pregnancy weight will be extracted from EMR, which was collected by women’s self-report. These indicators will be used to calculate BMI for initial assessment of eligibility.

We will use questionnaires to measure women’s behaviors (food intake, dietary behavior, physical activity, sedentary behavior, average steps during the last week), and other potential mediators of the intervention effect (e.g., attitude and knowledge about EGWG, stage of readiness) for behavioral change related to gestational weight control, social support, self-efficacy, frequency of weighing at three timepoints.

The cardiometabolic markers measured in women’s laboratory tests included levels of glucose, total cholesterol, triglycerides, high-density lipoprotein (HDL), low-density lipoprotein (LDL) during routine prenatal check-ups in the early, middle and late stages of pregnancy, and results of a 75 g 2-hour oral glucose tolerance test (OGTT) will be obtained from EMR. Women’s fasting blood samples at 8^+ 0^ to 13^+ 6^, 24^+ 0^ to 30^+ 6^, and 34^+ 0^ to 37^+ 6^ weeks’ gestation will be collected to measure levels of insulin, HbA1c, et al. Urine samples at three appointments and cord blood at birth will be collected for further analyses.

#### Measures about mother and infant 

women’s weight, blood pressure before delivery, infants’ gestational age at birth, birth weight, recumbent length at birth, Apgar scores, et al. will be obtained from EMR at one week after delivery. Maternal postpartum weight, and infants’ height, weight, head circumference at 42 days, 6 months and 1 year after delivery will be obtained through routine physical examination or telephone follow-up.

#### Sociodemographic characteristics and outcomes 

Participants’ sociodemographic characteristics including age, educational level, occupation, socio-economic status (health insurance, household income last year), smoking and alcohol consumption, medical and reproductive history will be assessed at the baseline survey at 8 to 13^+ 6^ weeks’ gestation. Other outcomes including sleep quality, quality of life, and mental health, et al.

### Adverse events

The perinatal adverse outcomes, including pregnancy loss, gestational diabetes, pregnancy-induced hypertension, preeclampsia, cesarean delivery, and preterm birth (before 37 weeks) will be obtained from the EMR.

### Fidelity/quality control of the intervention

Data collection and intervention are performed by two key members of the research team, who know the research protocol well. Two operation manuals according to each hospital’s routine perinatal care process have been developed for implementing and managing the intervention. The manuals describe in detail when, where and what the project staff should do in delivering the intervention. Both of the two project staffs will be required to perform the intervention according to the operation manuals.

During the implementation of this intervention, regular online meetings will be held to report the implementation process and record problems and good experiences will also be shared to facilitate the intervention in the future.

### Strategies to enhance clinical trial compliance and retention

To maximize compliance and retention in this study, we achieved the greatest support from the hospitals. The project staff will undertake the recruitment, survey and intervention in the obstetric clinic where all early pregnant women must come to have a clinical visit with the assistance of obstetricians and nurses. If participants are found not in compliance, a telephone call, Wechat messages, or face-to-face interview will be conducted to tell the importance and the current weight gain status to let the women continue to keep compliance by using motivational interviewing. Additional strategies will include newsletters underscoring key points to do during the intervention.

### Outcomes

*The primary outcomes* of this study are the total GWG up to 32^+ 0^−35^+ 6^ weeks’ gestation and the GWG prior to the OGTT screening at 24^+ 0^−27^+ 6^ weeks’ gestation. Both will be calculated by subtracting the pre-pregnancy weight from the weight measured at the respective time points.

*The secondary outcomes *include rate of GWG per week. Weekly rate of GWG will be defined as GWG divided by the number of weeks between the date of LMP and the midterm survey (24^+ 0^ to 27^+ 6^ weeks’ gestation) and the terminal survey (32^+ 0^ to 35^+ 6^ weeks’ gestation). Weekly rate of GWG between study clinical visits at 8^+ 0^ weeks’ and 27^+ 6^ weeks’ gestation, and at 8^+ 0^ and 35^+ 6^ weeks’ gestation will be calculated by dividing the amount of weight gain between visits by the weeks of gestation between visits. The proportion of women who exceed the 2009 IOM guidelines’ weekly rate of GWG will also be assessed, and it was defined as being above the upper limit of the first trimester (2.0 kg) plus the upper limit of the weekly rate (0.33 kg/week for overweight with BMI ranging from 24.0 to 28.0 kg/m^2^, 0.27 kg/week for obesity with BMI ≥ 28 kg/m^2^), times the number of weeks in the second and third trimesters.

Other secondary outcomes include incidence of GDM diagnosed according to IADPSG criteria (FPG ≥ 5.1 mmol/L, and/or 1-h blood glucose ≥ 10.0 mmol/L, and/or 2-h blood glucose ≥ 8.5 mmol/L), rate of preeclampsia/gestational hypertension (preeclampsia was defined according to the criteria of the ACOG, i.e. systolic blood pressure of ≥ 140 mmHg or diastolic blood pressure of ≥ 90 mmHg occurring after 20 weeks of gestation in a previously normotensive woman combined with new-onset proteinuria of ≥ 0.3 g/24 h. Gestational hypertension was defined similarly but without the presence of proteinuria.), incidence of cesarean delivery, absolute/mean infant birth weight, incidence of macrosomia (with infant birth weight ≥ 4000 g), and absolute/mean Apgar scores at 1–5 min (Apgar scores at 1–5 min evaluate the baby’s heart rate, respiratory effort, muscle tone, reflex irritability, and color. Each category is scored from 0 to 2, and the total score ranges from 0 to 10. A higher Apgar score indicates that the newborn is in good health.).

In addition, pre-specified secondary outcomes also include changes of blood pressure, body fat percentage (%), fat free mass (kg), muscle mass (kg), attitude and knowledge, dietary intake, physical activity, sleep quality, quality of life, self-efficacy score, stage of change related to gestational weight control behavior, and incidence of depression and anxiety, which will be assessed from baseline (8^+ 0^−13^+ 6^ weeks) to midterm (24^+ 0^−27^+ 6^ weeks), and from baseline (8–14 weeks) to terminal (32^+ 0^−35^+ 6^ weeks). Incidence of postpartum weight retention, absolute/mean offspring weight, height, and head circumference will also be evaluated at 42 days, 6 months, and 12 months after delivery.

### Statistical analyses

Baseline characteristics of participants will be reported by using descriptive statistics. Statistical analyses will be based on the intention-to-treat principle, and include all women recruited with BMI ≥ 24 kg/m^2^. Multiple linear regression models/Generalized linear mixed models will be used to examine the primary and secondary outcomes at 24^+ 0^−27^+ 6^ and 32^+ 0^−35^+ 6^ gestational weeks, at delivery, at 42 d, 6 m, and 12 m after delivery, and the models will be adjusted for the clustering effect and the baseline outcome values. Chi-squared tests will be used to compare conditions on the proportion of women who exceed the 2009 IOM guidelines’ weekly rate of GWG, and other dichotomous outcomes. We will also analyze whether the outcome disparities between the intervention and control groups vary by hospitals, BMI status before pregnancy, etc. All results will be considered statistically significant as two-side *P* < 0.05. Statistical analyses will be conducted by using R software 4.4.1.

## Discussion

Excessive gestational weight gain (EGWG) has been shown to amplify the effect of overweight or obesity on the risk of GDM, macrosomia, large for gestational age(LGA), and adverse health outcomes during childhood and adolescence, such as obesity, early puberty, and type 2 diabetes [7]. Preventing EGWG among high-risk pregnant women may have the greatest long-term effects in promoting the health of both mothers and offspring. Although a large amount of EGWG intervention study has been carried out, there was considerable heterogeneity among different intervention studies, including type, intensity of the intervention provided, the amount and type of face-to-face contact provided within the intervention, and whether it was performed based on mobile health or not, etc [40]. There is an urgent need for a widely recognized mobile health-based intervention models for the effective prevention of EGWG, especially for high-risk pregnant women such as overweight or obesity before pregnancy.

The HGWG-pro trial is designed to advance our knowledge of mobile health-based lifestyle interventions to optimize GWG and other health outcomes in pregnant women who are overweight or obese, as well as in their offspring. Strengths of the trial design include recruitment from two regions with different economic levels, the guidance of the transtheoretical model, the support with mobile health technologies (APP and wristband), focusing on changing 8 unhealthy dietary behaviors. Data collection strengths include rigorous assessment of food frequency, assessment of participants’ objective physical activity during pregnancy by collecting data through Wechat APP and wristband, collection of participants’ blood and urine samples at three different stages of pregnancy, as well as cord blood, to evaluate the cardiometabolic profile and creation of a biorepository for future study. The 12-month follow-up data will facilitate determining whether potential improvements in GWG are associated with improvements in postpartum weight retention and child growth and development indicators. It can serve as the first examples of a rigorously designed and evaluated EGWG prevention program improving dietary and exercise behaviors in China.

The HGWG-pro study is uniquely positioned to address the critical issue of poor adherence to intervention components, a persistent limitation in previous studies. Through the established collaborative partnerships with local hospitals, we have strategically integrated the weight management intervention into prenatal care procedures. This innovative approach embeds the intervention components within the standard obstetric examination workflow, thereby enhancing participant adherence by minimizing additional burdens on pregnant women. The rigorous quality control measures and close coordination with hospital staff ensure consistent implementation and monitoring of the intervention protocol throughout the pregnancy care continuum.

## Data Availability

Research data are not publicly available but can be obtained from the corresponding author on request.

## Abbreviations

ACOG: American College of Obstetricians and Gynecologists
BMI: Body mass index
EGWG: Excessive gestational weight gain
FPG: Fasting plasma glucose
EMR: Electronic medical record
FFQ: Food frequency questionnaire
GDM: Gestational diabetes mellitus
GI: Glycemic index
GLOW: Gestational Weight Gain and Optimal Wellness
GSES: General Self-Efficacy Scale
GWG: Gestational weight gain
HDL: High-density lipoprotein
HGWG-pro: Healthy Gestational Weight Gain program
IADPS: International Association of Diabetes and Pregnancy Study Group
IOM: Institute of Medicine
IPAQ-SF: International Physical Activity Questionnaire-Short Form
LDL: Low-density lipoprotein
LGA: Large for gestational age
LMP: Last menstrual period
MET: Metabolic equivalent
MSSS: Maternity Social Support Scale
OGTT: oral glucose tolerance test
PAR-Q: Physical Activity Readiness Questionnaire
PHQ-9: Patient Health Questionnaire
PSQI: Pittsburgh Sleep Quality Index
SAS: Self Rating Anxiety Scale
SF-12: 12-Item Short Form Survey
TTM: Transtheoretical model and stages of change

## References

[1] Deputy NP, Sharma AJ, Kim SY, Hinkle SN. Prevalence and characteristics associated with gestational weight gain adequacy. Obstet Gynecol. 2015;125(4):773–81. 25751216 10.1097/AOG.0000000000000739 PMC4425284

[2] Restall A, Taylor RS, Thompson JMD, Flower D, Dekker GA, Kenny LC, et al. Risk factors for excessive gestational weight gain in a healthy, nulliparous cohort. J Obes. 2014;2014:148391. 24995130 10.1155/2014/148391 PMC4065732

[3] Bahri Khomami M, Teede HJ, Enticott J, O’Reilly S, Bailey C, Harrison CL. Implementation of antenatal lifestyle interventions into routine care. JAMA Netw Open. 2022;5(10):e2234870. 36197663 10.1001/jamanetworkopen.2022.34870 PMC9535535

[4] Borges MC, Clayton GL, Freathy RM, Felix JF, Fernandez-Sanles A, Soares AG, et al. Integrating multiple lines of evidence to assess the effects of maternal BMI on pregnancy and perinatal outcomes. BMC Med. 2024. 10.1186/s12916-023-03167-0. 38281920 10.1186/s12916-023-03167-0 PMC10823651

[5] Ma RCW, Voerman E, Santos S, Patro Golab B, Amiano P, Ballester F, Barros H, Bergström A, Charles M-A, Chatzi L, et al. Maternal body mass index, gestational weight gain, and the risk of overweight and obesity across childhood: an individual participant data meta-analysis. PLoS Med. 2019;16(2):e1002744. 30742624 10.1371/journal.pmed.1002744 PMC6370184

[6] Goldstein RF, Abell SK, Ranasinha S, Misso M, Boyle JA, Black MH, et al. Association of gestational weight gain with maternal and infant outcomes. JAMA. 2017;317(21):2207–25. 28586887 10.1001/jama.2017.3635 PMC5815056

[7] Ferrara A. Translating research on diabetes and obesity in pregnancy into prevention: the 2019 Norbert Freinkel award lecture. Diabetes Care. 2020;43(11):2635–42. 33082243 10.2337/dci19-0040 PMC7576424

[8] Teede HJ, Bailey C, Moran LJ, Bahri Khomami M, Enticott J, Ranasinha S, et al. Association of antenatal diet and physical activity–based interventions with gestational weight gain and pregnancy outcomes. JAMA Intern Med. 2022. 10.1001/jamainternmed.2021.6373. 34928300 10.1001/jamainternmed.2021.6373 PMC8689430

[9] Bailey C, Skouteris H, Harrison CL, Hill B, Thangaratinam S, Teede H, et al. A comparison of the cost-effectiveness of lifestyle interventions in pregnancy. Value Health. 2022;25(2):194–202. 35094792 10.1016/j.jval.2021.07.013

[10] Cantor AG, Jungbauer RM, McDonagh M, Blazina I, Marshall NE, Weeks C, Fu R, LeBlanc ES, Chou R. Counseling and behavioral interventions for healthy weight and weight gain in pregnancy. JAMA. 2021;325(20):2094–109. 34032824 10.1001/jama.2021.4230

[11] Vesco KK, Karanja N, King JC, Gillman MW, Leo MC, Perrin N, McEvoy CT, Eckhardt CL, Smith KS, Stevens VJ. Efficacy of a group-based dietary intervention for limiting gestational weight gain among obese women: a randomized trial. Obes (Silver Spring). 2014;22(9):1989–96. 10.1002/oby.20831 PMC4407817 25164259

[12] Peaceman AM, Clifton RG, Phelan S, Gallagher D, Evans M, Redman LM, et al. Lifestyle interventions limit gestational weight gain in women with overweight or obesity: LIFE-Moms prospective meta-analysis. Obesity. 2018;26(9):1396–404. 30230252 10.1002/oby.22250 PMC6148360

[13] Ferrara A, Hedderson MM, Brown SD, Ehrlich SF, Tsai A-L, Feng J, Galarce M, Marcovina S, Catalano P, Quesenberry CP. A telehealth lifestyle intervention to reduce excess gestational weight gain in pregnant women with overweight or obesity (GLOW): a randomised, parallel-group, controlled trial. Lancet Diabetes Endocrinol. 2020;8(6):490–500. 32445736 10.1016/S2213-8587(20)30107-8 PMC8886506

[14] Raab R, Geyer K, Zagar S, Hauner H. App-supported lifestyle interventions in pregnancy to manage gestational weight gain and prevent gestational diabetes: scoping review. J Med Internet Res. 2023. 10.2196/48853. 37948111 10.2196/48853 PMC10674147

[15] McGovern L, O’Toole L, Houshialsadat Z, O’Reilly SL. Women’s perspectives on mHealth behavior change interventions for the management of overweight, obesity, or gestational diabetes: a qualitative meta-synthesis. Obes Rev. 2024. 10.1111/obr.13761. 38733067 10.1111/obr.13761

[16] Redman LM, Gilmore LA, Breaux J, Thomas DM, Elkind-Hirsch K, Stewart T, Hsia DS, Burton J, Apolzan JW, Cain LE, et al. Effectiveness of SmartMoms, a novel eHealth intervention for management of gestational weight gain: randomized controlled pilot trial. JMIR Mhealth Uhealth. 2017;5(9):e133. 28903892 10.2196/mhealth.8228 PMC5617906

[17] Zhang Y, Wang L, Yang W, Niu D, Li C, Wang L, et al. Effectiveness of low glycemic index diet consultations through a diet glycemic assessment app tool on maternal and neonatal insulin resistance: a randomized controlled trial. JMIR Mhealth Uhealth. 2019;7(4):e12081. 30998227 10.2196/12081 PMC6503641

[18] Kennelly MA, Ainscough K, Lindsay K, Gibney E, Mc Carthy M, McAuliffe FM. Pregnancy, exercise and nutrition research study with smart phone app support (Pears): study protocol of a randomized controlled trial. Contemp Clin Trials. 2016;46:92–9. 26625980 10.1016/j.cct.2015.11.018

[19] Louise J, Poprzeczny AJ, Deussen AR, Vinter C, Tanvig M, Jensen DM, et al. The effects of dietary and lifestyle interventions among pregnant women with overweight or obesity on early childhood outcomes: an individual participant data meta-analysis from randomised trials. BMC Med. 2021. 10.1186/s12916-021-01995-6. 34074261 10.1186/s12916-021-01995-6 PMC8170974

[20] Wang M, Xu S, Liu W, Zhang C, Zhang X, Wang L, et al. Prevalence and changes of BMI categories in China and related chronic diseases: cross-sectional National Health Service Surveys (NHSSs) from 2013 to 2018. Eclin Med. 2020. 10.1016/j.eclinm.2020.100521. 10.1016/j.eclinm.2020.100521 PMC7492818 32984787

[21] China NBoSo. https://data.stats.gov.cn/search.htm?s=GDP. In.; 2025.

[22] Barba C, Cavalli-Sforza T, Cutter J, Darnton-Hill I, Deurenberg P, Deurenberg-Yap M, Gill T, James P, Ko G, Miu AH, et al. Appropriate body-mass index for Asian populations and its implications for policy and intervention strategies. Lancet. 2004;363(9403):157–63. 14726171 10.1016/S0140-6736(03)15268-3

[23] Simmons D, Devlieger R, van Assche A, Jans G, Galjaard S, Corcoy R, et al. Effect of physical activity and/or healthy eating on GDM risk: the DALI lifestyle study. J Clin Endocrinol Metab. 2017;102(3):903–13. 27935767 10.1210/jc.2016-3455 PMC5460688

[24] China, NHCotPsRo. Standard of recommendation for weight gain during pregnancy period. In. Beijing, China: National Health Commission of the People’s Republic of China; 2022.

[25] Society CN. Dietary Guidelines for Chinese Residents. Beijing: People’s Medical Publishing House; 2022.

[26] Shub A, Huning EYS, Campbell KJ, McCarthy EA. Pregnant women’s knowledge of weight, weight gain, complications of obesity and weight management strategies in pregnancy. BMC Res Notes. 2013;6:278. 23866845 10.1186/1756-0500-6-278 PMC3726511

[27] Bookari K, Yeatman H, Williamson M. Australian pregnant women’s awareness of gestational weight gain and dietary guidelines: opportunity for action. J Pregnancy. 2016;2016:8162645. 26881080 10.1155/2016/8162645 PMC4736585

[28] Gao J, Fei J, Jiang L, Yao W, Lin B, Guo H. ASSESSMENT OF THE REPRODUCIBILITY AND VALIDITY OF A SIMPLE FOOD-FREQUENCY QUESTIONNAIRE USED IN DIETARY PATTERNS STUDIES. Acta Nutr Sin. 2011;33(5):452–6.

[29] Simmons D, Jelsma JGM, Galjaard S, Devlieger R, van Assche A, Jans G, Corcoy R, Adelantado JM, Dunne F, Desoye G, et al. Results from a European multicenter randomized trial of physical activity and/or healthy eating to reduce the risk of gestational diabetes mellitus: the DALI lifestyle pilot. Diabetes Care. 2015;38(9):1650–6. 26112044 10.2337/dc15-0360

[30] Simmons LA, Phipps JE, Overstreet C, Smith PM, Bechard E, Liu S, et al. Goals for reaching optimal wellness (GROWell): a clinical trial protocol of a digital dietary intervention for pregnant and postpartum people with prenatal overweight or obesity. Contemp Clin Trials. 2022. 10.1016/j.cct.2021.106627. 34813963 10.1016/j.cct.2021.106627 PMC9044978

[31] Macfarlane DJ, Lee CCY, Flo EY, Chan KL, Chan DTS. Reliability and validity of the Chinese version of IPAQ (short, last 7 days). J Sci Med Sport. 2007;10(1):45–51. 16807105 10.1016/j.jsams.2006.05.003

[32] Buysse DJ, Reynolds CF, Monk TH, Berman SR, Kupfer DJ. THE PITTSBURGH SLEEP QUALITY INDEX - A NEW INSTRUMENT FOR PSYCHIATRIC PRACTICE AND RESEARCH. Psychiatry Res. 1989;28(2):193–213. 2748771 10.1016/0165-1781(89)90047-4

[33] Ware JE, Kosinski M, Keller SD. A 12-item short-form health survey - construction of scales and preliminary tests of reliability and validity. Med Care. 1996;34(3):220–33. 8628042 10.1097/00005650-199603000-00003

[34] Kroenke K, Spitzer RL, Williams JBW. The PHQ-9 - validity of a brief depression severity measure. J Gen Intern Med. 2001;16(9):606–13. 11556941 10.1046/j.1525-1497.2001.016009606.x PMC1495268

[35] Wang W, Bian Q, Zhao Y, Li X, Wang W, Du J, Zhang G, Zhou Q, Zhao M. Reliability and validity of the Chinese version of the patient health questionnaire (PHQ-9) in the general population. Gen Hosp Psychiatry. 2014;36(5):539–44. 25023953 10.1016/j.genhosppsych.2014.05.021

[36] Zung WWK. RATING INSTRUMENT FOR ANXIETY DISORDERS. Psychosomatics. 1971;12(6):371–. 5172928 10.1016/S0033-3182(71)71479-0

[37] Webster J, Linnane JWJ, Dibley LM, Hinson JK, Starrenburg SE, Roberts JA. Measuring social support in pregnancy:: can it be simple < i > and meaningful? Birth-Issues in perinatal care 2000, 27(2):97–101. 10.1046/j.1523-536x.2000.00097.x 11251486

[38] Zhang JX, Schwarzer R: Measuring optimistic self-beliefs - A Chinese adaptation of the general self-efficacy scale. Psychologia 1995;38(3):174–181.

[39] Cheung SK, Sun SYK. Assessment of optimistic self-beliefs: further validation of the Chinese version of the general self-efficacy scale. Psychol Rep. 1999;85(3):1221–4. 10710976 10.2466/pr0.1999.85.3f.1221

[40] Dodd JM, Deussen AR, Poprzeczny AJ, Slade LJ, Mitchell M, Louise J. Investigating discrepancies in findings between rigorous randomized trials and meta-analyses evaluating pregnancy interventions to limit gestational weight gain. Obes Rev 2024; 25(12). 10.1111/obr.13826 39363588

